# The energetics of cellular life transitions

**DOI:** 10.1093/lifemeta/load051

**Published:** 2023-12-27

**Authors:** Anna S Monzel, Michael Levin, Martin Picard

**Affiliations:** Department of Psychiatry, Division of Behavioral Medicine, College of Physicians and Surgeons, Columbia University Irving Medical Center, New York, NY 10032, United States; Department of Biology, Tufts University, Medford, MA 02155, United States; Allen Discovery Center at Tufts University, Medford, MA 02155, United States; Wyss Institute for Biologically Inspired Engineering, Harvard University, Boston, MA 02115, United States; Department of Psychiatry, Division of Behavioral Medicine, College of Physicians and Surgeons, Columbia University Irving Medical Center, New York, NY 10032, United States; Department of Neurology, H. Houston Merritt Center, Columbia Translational Neuroscience Initiative, College of Physicians and Surgeons, Columbia University Irving Medical Center, New York, NY 10032, United States; New York State Psychiatric Institute, New York, NY 10032, United States; Robert N Butler Columbia Aging Center, Columbia University Mailman School of Public Health, New York, NY 10032, United States

**Keywords:** energy, mitochondria, signaling pathway, growth differentiation factor 15, energy balance, development

## Abstract

Major life transitions are always difficult because change costs energy. Recent findings have demonstrated how mitochondrial oxidative phosphorylation (OxPhos) defects increase the energetic cost of living and that excessive integrated stress response (ISR) signaling may prevent cellular identity transitions during development. In this perspective, we discuss general bioenergetic principles of life transitions and the costly molecular processes involved in reprograming the cellular hardware/software as cells shift identity. The energetic cost of cellular differentiation has not been directly quantified, representing a gap in knowledge. We propose that the ISR is an energetic checkpoint evolved to (i) prevent OxPhos-deficient cells from engaging in excessively costly transitions and (ii) allow ISR-positive cells to recruit systemic energetic resources by signaling via GDF15 and the brain.

Thermodynamic principles stipulate that without the input of energy, a system is doomed to either drift toward increasing entropy or to remain in its initial state. For this reason, any type of change must consume energy. In other words, change costs energy. But living cells and organisms constantly face the need for change, including energetically costly transitions from one cellular state to another, or from one position to another within the organism [1]. Bearing adequate resources, all developing cells and subpopulations of adult cells must, at some point, transition from their relatively undifferentiated pluripotent identity to some “terminal” specialized identity. Therefore, understanding the core subcellular mechanisms that monitor a cell’s energetic status and that also energetically sustain their challenging life transitions, has the potential to uncover biological principles that are the basis of human health, or that falter in diseases.

## Life transitions cost energy

**Figure 1:**
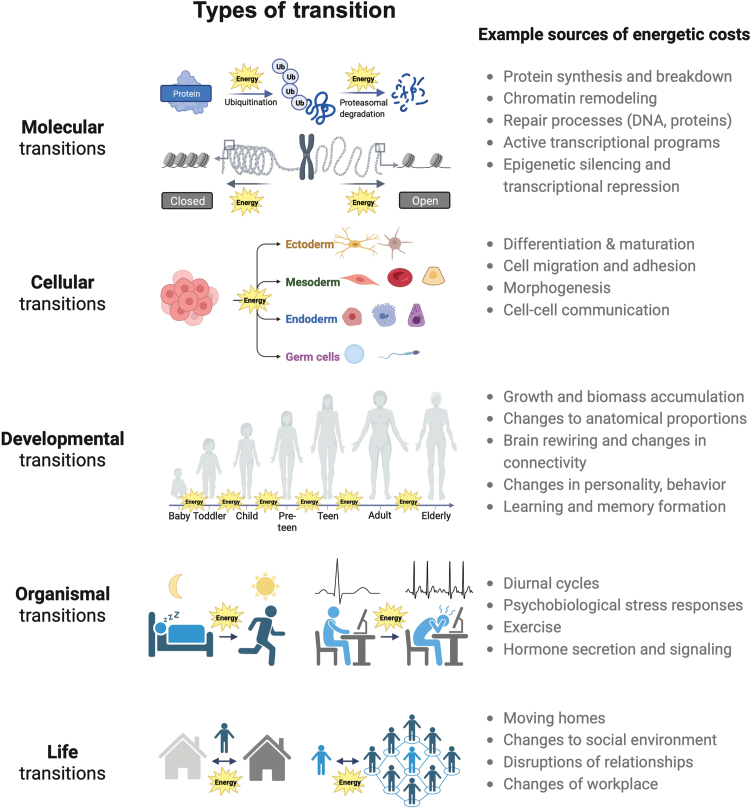
Energetically costly transitions occur at all levels of complexity. Molecular transitions involve the breakdown and synthesis of molecules, cellular transitions require changes in a cell’s hardware and software, developmental transitions involve organism-level changes in how the body develops from birth through pub erty and aging, organismal transitions relate to changes in the integrated state of the body–mind system, and life transitions involve changes within and outside an individual in relation to their environment. All transitions have a common denominator, namely the need for energy input required to effect change and undergo any type of transition.

Life transitions and identity changes are always difficult. This principle applies across levels of complexity: molecules rearrange or break down via endergonic chemical reactions, pluripotent cells transition from a stem-like state into a terminally differentiated identity, and complex conscious organisms are forced to transition from one developmental life stage to another [2]. The difficulty with transitions likely emerges from the activation energy barriers that must be overcome to achieve change (**Fig. 1**). In the same way that many enzymes require activation energy (e.g., ATP, NAD(P)H, and others) to power the molecular transition of substrate A into product B, the human mind and body require inputting energy to power all meaningful life transitions. For instance, major life transitions such as moving into a new home or changing one’s social relationship(s) all come with some degree of uncontrollability, leading to psychobiological stress [3]. The neuroendocrine stress responses engendered by the resulting uncomfortable psychological states consume considerable amounts of energy [4]. As a result, major life and organismal transitions must be energetically costly.

**Figure 2:**
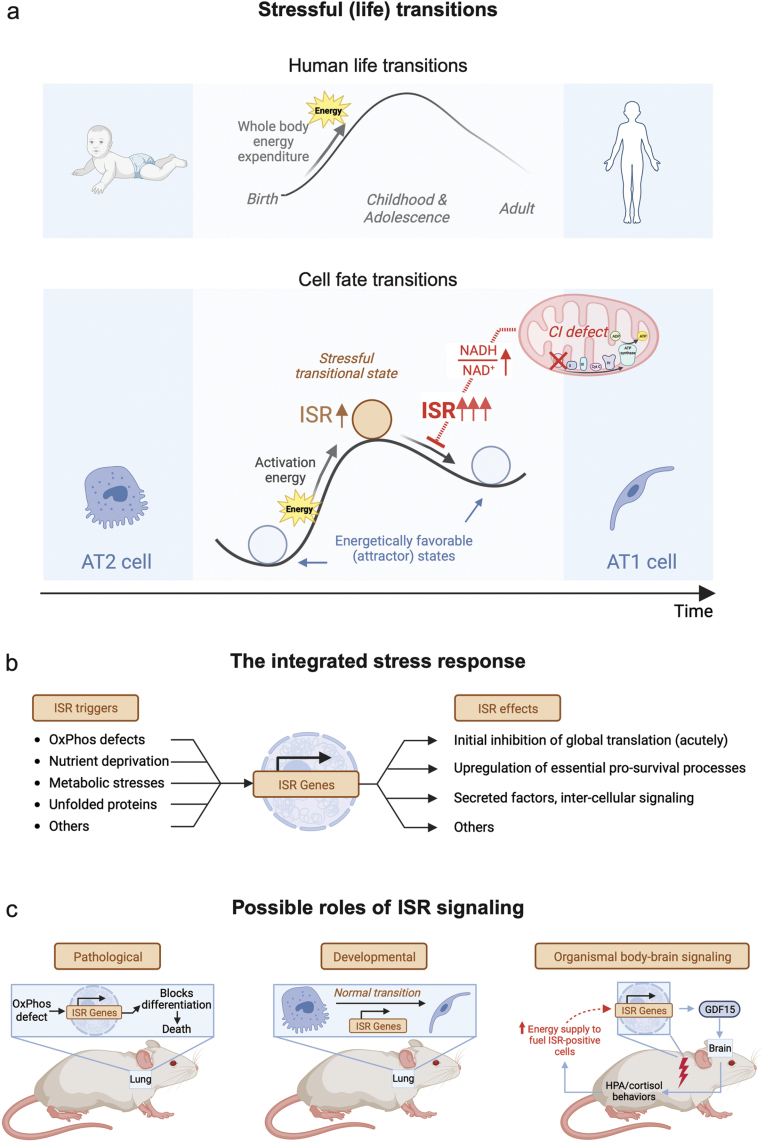
Stressful life transitions and the ISR. (a) Life transitions are energetically costly and therefore stressful, both for organism and for individual cells. As recently shown in the lung epithelium [6], the mitochondrial ISR is naturally upregulated in transitional mouse AT2-to-AT1 alveolar epithelial cells. The complex I defect in this lineage further amplifies ISR activation, which prevents cells from completing the developmental identity shift. (b) The ISR is triggered by primary mitochondrial OxPhos defects and metabolic stress caused by a variety of environmental and intrinsic stressors, mediated by NADH reductive stress. In turn, the ISR triggers several pro-survival signals, presumably to restore cellular homeostasis, which interferes with vital cellular processes including differentiation. (c) Three non-mutually exclusive interpretations of ISR involvement in health and disease: (Left) ISR activation can be triggered by severe OxPhos defects, interfere with differentiation, and lead to premature death, categorizing its function as pathological. (Middle) The ISR is mildly upregulated in normal cellular transition between the stem-like and differentiated states in mouse lung epithelial cells, suggesting that the ISR is a normal checkpoint to ensure the availability of sufficient energetic resources to proceed with differentiation and possibly other cellular behaviors. (Right) The involvement of ISR in organismal somato-cognitive signaling suggests that downstream products of the ISR, including the secreted metabokine GDF15, signal to recruit energetic resources in an attempt to rescue energetically challenged, ISR-positive cells and tissues. See text for additional details.

The most energetically costly period of human life—where whole-body energy expenditure normalized per unit of body mass is the highest—is between ages 1 and 15 [5]. This period is replete with changes as the infant develops an identity as a child and later transitions into adolescence and adulthood. Over this critical life stage, the body undergoes phases of rapid growth and biomass accumulation, as well as pruning and establishing billions of neural connections. In parallel, major psychobiological changes must occur as personality and the ego or “I” develop, occurring alongside life-defining anatomical, physiological, and hormonal changes (**Fig. 2a**, top). Interestingly, this costly developmental transition through adolescence is marked by the highest risk of onset for mental health disorders [7]. The individual vulnerability around this period of high energy demand suggests that competing demands for limited energetic resources could interfere with developmental processes, resilience, and health [8].

Below, we focus our discussion on life transitions at the cellular level. Similar to human life transitions, cellular maturation and identity changes operate under the same principles and face similarly costly challenges. But, cellular transitions occur at a scale amenable to the scientific scalpel of molecular biology and the precise mechanistic dissection it affords. Thus, understanding the energetic principles that guide these processes may yield new insights into the energetic forces and molecular pathways that monitor and constrain differentiation.

### Changes required for cellular and tissue-level transitions

One of the hallmarks of multicellular organisms is that the activity of individual cells is harnessed toward broader organismal goals. This includes creating complex anatomical structures and morphologies (i.e., morphospace) [9]. Starting from the fertilized egg, all descendants of that cell must differentiate, migrate, and cooperate with each other to eventually reach the possible region with configurations that correspond to the normal species-specific anatomy. Yet, after differentiation, cells keep changing and adapting in response to environmental cues. They are constantly actively monitoring their state, computing errors from their final position and function, and working to reduce those errors [10–12]. These energy-consuming actions require reprogrammability.

While genomes encode the specific hardware that each cell has access to (via the proteins specified by the DNA sequence and chromatin state), the behavior of each cell and the cell collective is driven by the physiological software that supervenes on the hardware [13, 14]. Note that the hardware-software analogy used here and in synthetic bioengineering and evolutionary developmental biology [15–17] refers not to the anthropomorphic origin of some (but no longer all [18]) software but rather refers to the fact that the exact same hardware (genomically determined cellular components) can give rise to numerous different outcomes. Just like the same physical computer hardware can run or execute vastly different software (text processing, video streaming, etc.).

A cell’s hardware includes the proteins encoded by the sequences of its genome that are epigenetically “open” and active or “closed” and repressed, plus the complex set of organelles, proteins, and molecules it contains at any given time [19]. The cell’s software includes the program(s) executed by the hardware, which comprise biomechanical [20–22], bioelectrical [23–25], and transcriptional [26–28] dynamics.

Software operations are driven not only by its inherited hardware but also by the metabolic and bioelectric cues that each cell receives from surrounding cells and the circulation and the specific configuration of its organelles and macromolecules. Software also integrates molecular, bioelectric, and metabolic memories of past exposures that shape signal transduction and stress responses [23]. Each of these programmable biological layers can process information, store memories, and interact with other layers. For example, bioelectric networks interface with numerous downstream transcriptional cascades as they determine organ shape and position [29], while gene-regulatory networks and pathways exhibit multiple different kinds of learning and memory [30, 31]. Targeting these components is an active area of research, complementing the decades of focus on genomic editing [32, 33].

On the scale of individual cells, differentiation from stem cells to specific fates involves decisions that shape the hardware and the subsequent activity and responses, which is seen in transcriptional, translational, and biophysical states. For example, each active gene comes at a substantial energetic cost [34]. The hardware of a differentiating cell is altered by actively repressing and silencing specific genes, while others are opened and transcribed. This gives rise to new proteins that must be energetically synthesized (4 ATP molecules per amino acid [35]), while others are actively degraded or secreted. The cell’s software is also reprogrammed by increasing or decreasing the resting plasma membrane potential, tweaking connections to surrounding cells, migrating, reconfiguring cytoplasmic constituents, and other non-random changes [23]. Thus, as cell fate transitions change a cell’s molecular identity—as well as its role within the organism—it requires widespread energy input to reprogram both the cellular hardware and software. As a result, reprogramming and cell fate transitions may represent one of the most stressful and energetically costly events that a cell has to go through.

### Stress and energy allocation

As introduced above, stress responses necessarily cost energy [4]. The term “stress” refers to any type of response that catalyzes change within a complex system, such as a transition from state Y to Z (e.g., resting to activated). Stress is often caused by external “stressors” that force a cell or organism to adapt and change its state. But stress is also triggered by internal processes, like a developmental trajectory that requires a cell to shift identity [36] or by the accumulation of damage during aging [37, 38].

Importantly, the costs of stress and other processes are generally cumulative. This means that the energy required to power hardware/software reprogramming during cellular transitions must come on top of what is required at baseline to prevent entropy and sustain life [39]. Thus, while a developing cell naturally expends energy to maintain integrity, it also must deploy energetically expensive molecular processes required to shift its identity toward its terminal state, adding to the energetic cost of life.

But, living systems possess a finite capacity to transform energy [40]. The interplay of limited energetic capacity with the competing demands of molecular processes leads to energy constraints both within cells [41] and organisms [42]. The limited energy budget means that not all cellular or physiological systems can be turned on at the same time. Moreover, some transformations are also not compatible and must be separated in time (e.g., catabolic and anabolic pathways). Therefore, by necessity and to preserve efficiency in a given physiological context, one state must be dominant over other ones.

Consequently, an evolutionarily-driven hierarchy of energy needs dictates which molecular system is prioritized over another one. For example, cells must prioritize the maintenance of vital membrane potential through ion pumps, and of protein synthesis by ribosomes, over the more acutely dispensable DNA and RNA synthesis by polymerases [43]. Similarly, at the scale of the mammalian body, blood flow to the so-called “vital” organs such as the brain, heart, liver, and kidneys is prioritized over digestive and connective tissues, particularly in times of (real or perceived) stress. Energy is a vital resource, distributed sparingly and economically between and within living cells.

### Mitochondria, energy, and questions around cell fate transitions

Aerobic mammalian cells derive most of their usable energy from the oxidative phosphorylation (OxPhos) system within mitochondria. The OxPhos system pulls and reacts hydrocarbon-derived electrons with oxygen to build a transmembrane charge that powers ATP synthesis. For this reason, mitochondria naturally play a permissive role in sustaining life and enabling all psychobiological life transitions [44]. By producing ATP on demand—that is, when ATP is hydrolyzed into ADP and inorganic phosphate—mitochondria essentially “keep the lights on”, enabling life’s vital operations. But an important question is whether beyond its permissive role, energy transformation by mitochondrial OxPhos also plays an instructive role in shaping major cellular transitions, including differentiation from a stem-like precursor cell type to a terminally differentiated state.

It is well recognized that by controlling apoptosis, mitochondria dictate the ultimate death transition [45, 46]. We also know that terminal cell differentiation across cell lineages and contexts acutely depends on [47–49] and is even temporally controlled [50, 51] by mitochondrial redox and metabolite signaling, fusion–fission dynamics, and energetics [52–56]. Thus, mitochondria unequivocally play an instructive role in contributing to fuel and regulate stressful cellular identity transitions.

However, one major outstanding question has persisted around the molecular mechanisms through which mitochondrial OxPhos defects are transduced to the nucleus, via so-called “retrograde signaling”. We recently reported that primary human fibroblasts with OxPhos defects—which exhibit nuclear stress responses—expend up to twice as much energy to stay alive under resting conditions [57]. OxPhos defects increase the cost of living, or energy expenditure, likely through multiple cellular, tissue-level, and physiological stress response mechanisms that promote hypermetabolism [58]. Similarly, glucocorticoid signaling alone (which is not intrinsically damaging) in primary fibroblasts also induces an “anticipatory” stress response that increases cellular energy expenditure by ~60% [59]. Based on the economy of cellular energy outlined above, the cost of stress responses may expectedly conflict with the energetic requirements of differentiation, as observed in patient-derived induced pluripotent stem cells where OxPhos defects interfered with cellular differentiation [60].

How are the competing energetic demands of responding to intrinsically stressful OxPhos defects reconciled with the energetic requirements of cell fate transition? Might energetic stress responses and cell fate transitions be incompatible? If they are incompatible, might this explain developmental failure and pathogenesis in animal models of OxPhos defects and patients with mitochondrial diseases?

## The ISR in mouse lung development

Recent research combining cell-type-specific genetic mitochondrial lesions, small-molecule inhibitors, and single-cell resolution mapping of cellular states in mice sheds light on the intersection of mitochondrial OxPhos defects and developmental cell fate transition [6]. In the study by Han *et al*., pulmonary alveolar cells with a defect in the mitochondrial OxPhos complex I subunit NADH dehydrogenase (ubiquinone) iron-sulfur protein 2 (Ndufs2) failed to transition toward their terminal identity, ultimately leading to respiratory failure and death. Importantly, transitioning cells employ a molecular mechanism acting as a checkpoint that inhibits a specific cell fate transition required for survival. This work raises several significant questions discussed below.

In a healthy lung, a population of partially committed alveolar epithelial stem cells (AT2) transitions to alveolar epithelial type 1 (AT1) cells, which transfer oxygen from the air and carbon dioxide from the blood to support respiration. Perturbing mitochondrial OxPhos complex I in this cell lineage prevented AT2 cells from acquiring their terminal AT1 identity [6]. Importantly, complex I deficiency did not prevent cellular transition by promoting stemness but rather by triggering a shift toward a “transitional” state somewhere between the AT2 and AT1 cellular states (Fig. 2a, bottom). The differentiation failure was neither driven by forced quiescence nor senescence, as OxPhos-deficient lungs had increased expression of the proliferation marker Ki67, although the transitional cell population was mildly positive for the senescence marker cyclin-dependent kinase inhibitor p21 (Cdkn1a) [6].

As transitional states involve extensive hardware and software remodeling, the “identity crisis” of transitional cells must bear substantial energetic costs to the cell. However, reliable data quantifying the energetic costs of cell fate transitions appear to be lacking in the literature, representing a major gap in knowledge. Nevertheless, cells naturally strive to acquire energetically “stable” or optimal states [36]. Under physiological conditions, driven by energy constraints, very few cells exist in such “unstable” and likely costly transitional states. This is confirmed by single-cell RNA sequencing datasets where transitional cells are rare (< 1%) occurrences [6]. Nevertheless, both in developing lung tissue and in injured lungs, naturally occurring transitional cells upregulate a key transcriptional stress pathway called the integrated stress response (ISR) [6, 61] (Fig. 2a, bottom). Thus, during normal development, transitional cells show evidence of stress in general and activation of ISR in particular.

### The mitochondrial ISR

The ISR, sometimes referred to as the mitochondrial ISR (ISRmt), is an evolutionarily conserved intracellular signaling network triggered in response to several environmental and cell-intrinsic stressors, including but not limited to nutrient deprivation, infections, misfolded proteins, and oxidative stresses [61, 62]. Upon stress signaling, phosphorylation of the translation initiation factor 2 (eIF2) signals through CHOP [CCAAT/enhancer binding protein (C/EBP) homology protein] (Ddit3) [63] and activating transcription factors Atf4 and Atf5 to upregulate the expression of several downstream genes [61, 64] (Fig. 2b). Other stress signal transduction cascades may also converge on the ISR [65]. The epistatic network of relationships among ISR genes is still being uncovered in different species and cell types.

One of the best-described ISR targets, growth differentiation factor 15 (GDF15), is transcribed and secreted into the extracellular space, making its way into the bloodstream to exert broad systemic signaling effects [66]. GDF15 concentration is elevated in the blood of patients with OxPhos defects [67, 68], in the media of cultured cells with genetic or pharmacological OxPhos defects [57], as well as under other metabolically challenging situations including pregnancy [69] and starvation [70]—making GDF15 a putative general marker of “energetic stress”. Another ISR target, fibroblast growth factor 21 (FGF21), is also systemically upregulated in human and animal mitochondrial OxPhos defects, where it contributes to systemic ISR signaling [71]. The ISR-GDF15 axis appears to act as a metabolic stress signaling network, which among other roles may exist to inform the brain of energetic/redox stress among somatic tissues [66]. Yet, the factors by which mitochondrial OxPhos defects engage the ISR *in vivo*, in different tissues and cell types, remain to be fully elucidated.

In mice, the ISR is triggered in affected tissues by mitochondrial OxPhos defects [64, 65, 72]. However, different OxPhos defects can trigger the ISR through different mechanisms [73, 74]. In addition, non-OxPhos mitochondrial stressors can also induce the ISR, including misfolded proteins in the mitochondrial matrix [75, 76] and iron deficiency via the mitochondrial death ligand signal enhancer 1 (DELE1) [77], indicating that several triggers and molecular pathways act upstream of the mitochondrial ISR.

### Reductive stress triggers the ISR

In the recent study of the developing mouse lung [6], deleting Ndufs2 in transitional epithelial cells triggered robust ISR activation, monitored by the expression of ~120 genes including *Gdf15*. Most of these genes were induced at levels above the expression levels naturally induced in healthy transitional lung epithelial cells, suggesting that the OxPhos defects tapped into the same pathway. However, in this case, the OxPhos defects amplified this pathway to a point where the ISR became maladaptive or pathological.

Both genetic and pharmacological OxPhos defects reliably activate *Gdf15* expression in mammalian cells, including in primary human fibroblasts [57]. Interestingly, pathological ISR activation in the developing lung was not driven by ATP deficiency. Rather, it was triggered by reductive stress, reflected by an increased NADH/NAD^+^ ratio, which is a natural consequence of mitochondrial complex I deficiency.

With normal OxPhos function, NADH donates its electron to complex I, oxidizing it back to NAD^+^. It was previously shown in myoblasts that oxidizing cytosolic NADH to NAD^+^ is sufficient to prevent ISR activation in complex I-deficient cells [73]. Recent work also confirmed that selectively inducing NADH reductive stress, without OxPhos deficiency, is sufficient to activate the ISR [78]. Han *et al*. extended these findings to the *in vivo* developing lung as they demonstrated that alleviating reductive stress by enzymatically oxidizing NADH prevented pathological alveolar ISR activation, thereby normalizing lung development [6]. Complex I-deficient mice protected from cytoplasmic reductive stress by overexpression of the yeast NADH dehydrogenase Ndi1 developed normally for up to 25 months.

In the context of monitoring costly cellular transitions, the NADH/NAD^+^ ratio is naturally a major driving force. The cellular energy landscape required to sustain stressful and costly transitions involves not only the phosphorylation potential (ATP/ADP/AMP) that powers several enzymatic reactions but also the redox potential (NADH/NAD^+^) that powers and regulates perhaps an equal, if not larger, number of molecular transitions [79]. The central dependence of so many reactions on NAD(P)H/NAD(P)^+^ ratio may account for why it has evolved—perhaps preferentially in some cell types—as a sufficient and perhaps most potent trigger of the ISR.

## Physiological roles of the ISR

Whereas some of the triggers of the ISR are relatively well defined in some contexts, what have remained less well established are the functional consequences of the ISR. Why did the ISR evolve? And what role does the ISR play in normal development and physiology, and in the pathophysiology of primary mitochondrial disorders and other diseases? Below, we summarize three possible, non-mutually exclusive interpretations.

### The ISR as pathology

The first and simplest interpretation is that the ISR is mostly activated under pathological conditions, playing little role in normal physiology. Several *in vitro* and *in vivo* studies have shown that primary disease-causing mitochondrial OxPhos defects activate the ISR [57, 64, 72, 73, 80], establishing that the ISR is active in non-homeostatic metabolic environments (reviewed in [62]). Upregulated under pathological conditions, the ISR places affected cells into a pro-survival state, likely as an attempt to re-establish cellular homeostasis [61]. Non-essential and costly cellular processes are put on hold (e.g., initial ISR-dependent inhibition of translation), while pro-survival essential processes are upregulated.

Direct support for the view that the ISR is “pathological” comes from the demonstration that inhibiting the ISR in the presence of the OxPhos complex I defects increases survival. In lung epithelial cells, ISR activation contributes to pathophysiology and mortality [6]. By serving as an inflexible cellular checkpoint, ISR activation in OxPhos-deficient developing alveolar epithelial cells appears to prevent the transition of AT2 cells into AT1 cells, without which the lung is not functional, and animals are not viable (Fig. 2c, left). Similarly, in mice with OxPhos-deficient skeletal muscles caused by mtDNA deletion, deleting FGF21 rescues some aspects of the pathological phenotype, suggesting that secreted ISR products contribute to pathophysiology in mice [71].

### The ISR as a cellular developmental checkpoint

A second related developmental proposition is that robust ISR activation evolved to prevent energetically compromised cells from engaging in excessively costly cell fate transitions. Initiating an energetically challenging transition without the assurance that sufficient resources are present could result in cell death, compromising animal survival. This is supported by the complete inhibition of AT2-to-AT1 transition in ISR-induced Ndufs2 knockout cells [6]. Normal developmental or regenerative transitions induce the ISR, but at lower levels that do not inhibit AT2-to-AT1 transitions (Fig. 2c, middle). In this case, the ISR may be triggered to prevent costly cellular commitment that could be fatal, and therefore detrimental to the organism.

This dual role of ISR upregulation in cellular differentiation was also demonstrated in a model of a rare genetic skeletal disorder, in which endoplasmic reticulum (ER) stress upregulated the ISR in hypertrophic chondrocytes [81]. Also, in this model, ISR-positive cells survived but transitioned back into a “juvenile” state. Importantly, *Atf4* was also expressed during normal (embryonic) development in differentiating chondrocytes. Inhibiting the ISR rescued skeletal abnormalities but had no impact on normal development of healthy (wild-type) mice [81]. These findings suggest that the ISR may be normally upregulated by default during development, possibly as a way of heightening the cell’s sensitivity to potential energetic stress. Supporting this view, other studies suggest that the ISR is activated preceding energetically demanding cellular expansion in activated T lymphocytes [82]. Iron deficiency can also trigger the ISR [83]. Whether stressors other than OxPhos also trigger the ISR to inhibit cell fate transitions remains unclear. More work is needed to understand the role of ISR activation during normal cellular differentiation and embryonic development.

### The ISR as a brain–body signaling mechanism

Let us remember that all mammalian nuclear programs and signaling pathways, including the ISR, must have evolved to optimize the fitness of the organism. The organism, not single cells, is the ultimate unit of natural selection. Therefore, we likely will approach the most accurate physiological interpretation of the ISR if we manage to understand its effects beyond the confine of the cell, in the context of the organism.

The third potential interpretation is that the ISR is a cellular-level sensor evolved to inform the organism of somatic energetic stress. In animals, ISR-mediated GDF15 secretion from affected tissues, including skeletal muscles [84] and adipocytes [85] appears to contribute to accelerating physiological processes, increasing whole-body energy expenditure [86]. OxPhos-deficient cells produce GDF15 that elevates the rate of energy transformation across the whole organism and likely plays other roles related to energy balance [62]. Intuitively, cells with energy production defects would be expected to adopt or deploy energy conservation strategies. However, as noted above, mitochondrial OxPhos defects increase energy expenditure to produce a state of cellular hypermetabolism [57]. In fact, OxPhos defects cause hyperactive physiology at multiple levels: OxPhos-deficient individuals secrete more catecholamines, have higher resting heart rate, grow more capillaries around OxPhos-deficient myofibers, and produce exaggerated cardiovascular and ventilatory responses to mild physical challenges [58, 87, 88]. Collectively, we can understand these hyperkinetic responses as mechanisms that cooperate to increase energy delivery to OxPhos-deficient, ISR-positive cells, which behave and signal as if energy starved.

Based on these facts, we reason that the physiological purpose of these ISR-driven hyperactive systems is to ensure the availability of energy resources (oxygen, carbon substrates) to all cells within the organism. The brain plays a major role in ensuring that cells and organs cooperate toward the same goal—the survival of the organism. Thus, the brain naturally harbors the cognate GDF15 receptor GFRAL (GDNF family receptor a–like) [89], allowing it to sense circulating GDF15, a major endocrine output of the ISR [66]. In mice, GDF15 also appears to recruit the glucose-releasing and hyperglycemia-promoting hormone corticosterone, further mobilizing energetic resources to fuel energy-starved ISR-positive and GDF15-secreting cells and tissues [90]. Thus, cells may use the ISR gene program and its secreted products to call in additional energetic resources from surrounding cells and organs to support them through challenging transitional times (Fig. 2c, right). But in OxPhos-deficient cells, the ISR appears permanently activated, the chronicity of which likely contributes to the pathological effects of the ISR.

The true function of the ISR is likely a dynamic combination of these propositions, expressed differently depending on the developmental context, cell type, stress exposure, and metabolic state of the organism.

## Conclusion

Life transitions are inherently “stressful” because they cost energy. OxPhos defects appear to increase the cost of life in cells, animals, and people [57, 58]. This likely occurs because cells that contain OxPhos-deficient mitochondria must bend their metabolism and behaviors out of shape, forced to induce and utilize less optimal metabolic strategies to survive. But in turn, the costly compensations that actively push them away from their optimal bioenergetic state require activating compensatory stress response pathways. These include but are not limited to the ISR. The ISR itself triggers costly genetic programs leading to metabokine protein synthesis, secretion, cell–cell signaling, and even somato-cognitive (i.e., body–brain) signaling that conveys systemic energetic distress to the brain [66].

Recent research shows that in the context of mitochondrial complex I-related OxPhos defects, the ISR can interfere with and prevent stressful cell fate transitions in mouse lung epithelial cells, resulting in death [6]. Building on previous [73] and recent work [78], the new findings cement the notion that reductive stress (elevated NADH/NAD^+^ ratio) is a necessary and sufficient ISR trigger in this *in vivo* context. Importantly, we also learn that normal developmental cell fate transitions involve ISR activation, suggesting that the ISR is a physiologically meaningful rheostat of stress signaling induced as part of normal, albeit challenging, cellular life transitions.

Each cell must operate as an information processing system monitoring its capacity to engage in costly life transitions. The new single-cell molecular details around ISR biology [6], interpreted through the lens of an economy of energy [39, 42, 91], suggests that the ISR is a central circuit of the cellular and mitochondrial information processing system [92] that weighs bioenergetic demand and supply.

But several questions arise. Does the ISR play the same “checkpoint” role in all cell types, across species? At the scale of an individual, do stressful and energetically costly lifespan psychobiological transitions also trigger the ISR or other energy monitoring pathways to steer the organism toward the most optimal path to adaptation? Can our understanding of ISR biology be harnessed to assist cells and individuals in reaching their full developmental and health potential?

Mitochondrial stress signaling is a burgeoning field replete with inspiring questions heavy with health implications. Addressing these questions across scales of complexity—from organelle to organism—requires that we integrate molecular energy sensing mechanisms within the brain-body and psychobiological systems that shape development and aging trajectories. Increasingly precise technologies and versatile approaches to probe stress responses across time and space make this a particularly exciting time for biology. Our ability to leverage new technologies informed by bioenergetic principles promises to produce increasingly more accurate models of health and development.
